# Longitudinal single-cell transcriptomics reveals distinct patterns of recurrence in acute myeloid leukemia

**DOI:** 10.1186/s12943-022-01635-4

**Published:** 2022-08-19

**Authors:** Yanan Zhai, Prashant Singh, Anna Dolnik, Peter Brazda, Nader Atlasy, Nunzio del Gaudio, Konstanze Döhner, Hartmut Döhner, Saverio Minucci, Joost Martens, Lucia Altucci, Wout Megchelenbrink, Lars Bullinger, Hendrik G. Stunnenberg

**Affiliations:** 1 Department of Precision Medicine, University of Campania “Luigi Vanvitelli”, Vico L. De Crecchio 7, 80138 Naples, Italy; 2 Prinses Maxima Centrum, Heidelberglaan 25, 3584 CS Utrecht, The Netherlands; 3 Department of Molecular Biology, Faculty of Science, Radboud Institute for Molecular Life Sciences, Radboud University, Nijmegen, the Netherlands; 4 Medical Department, Division of Hematology, Oncology, and Cancer Immunology, Charité – Universitätsmedizin Berlin, corporate member of Freie Universität Berlin, Humboldt-Universität Zu Berlin, Berlin Institute of Health, Berlin, Germany; 5 German Cancer Consortium (DKTK), German Cancer Research Center (DKFZ), Heidelberg, Germany; 6 Department of Internal Medicine III, University Hospital of Ulm, Ulm, Germany; 7 Department of Experimental Oncology, European Institute of Oncology IRCCS, Milan, EO Italy; 8 Institute of Molecular Biology and Genetics, BIOGEM, Ariano Irpino, AV Italy

**Keywords:** Acute myeloid Leukemia, Single-cell RNA sequencing, Recurrence, Leukemic stem cells, Genome analysis

## Abstract

**Background:**

Acute myeloid leukemia (AML) is a heterogeneous and aggressive blood cancer that results from diverse genetic aberrations in the hematopoietic stem or progenitor cells (HSPCs) leading to the expansion of blasts in the hematopoietic system. The heterogeneity and evolution of cancer blasts can render therapeutic interventions ineffective in a yet poorly understood patient-specific manner. In this study, we investigated the clonal heterogeneity of diagnosis (Dx) and relapse (Re) pairs at genetic and transcriptional levels, and unveiled the underlying pathways and genes contributing to recurrence.

**Methods:**

Whole-exome sequencing was used to detect somatic mutations and large copy number variations (CNVs). Single cell RNA-seq was performed to investigate the clonal heterogeneity between Dx-Re pairs and amongst patients.

**Results:**

scRNA-seq analysis revealed extensive expression differences between patients and Dx-Re pairs, even for those with the same -presumed- initiating events. Transcriptional differences between and within patients are associated with clonal composition and evolution, with the most striking differences in patients that gained large-scale copy number variations at relapse. These differences appear to have significant molecular implications, exemplified by a DNMT3A/FLT3-ITD patient where the leukemia switched from an AP-1 regulated clone at Dx to a mTOR signaling driven clone at Re. The two distinct *AML1-ETO* pairs share genes related to hematopoietic stem cell maintenance and cell migration suggesting that the Re leukemic stem cell-like (LSC-like) cells evolved from the Dx cells.

**Conclusions:**

In summary, the single cell RNA data underpinned the tumor heterogeneity not only amongst patient blasts with similar initiating mutations but also between each Dx-Re pair. Our results suggest alternatively and currently unappreciated and unexplored mechanisms leading to therapeutic resistance and AML recurrence.

**Supplementary Information:**

The online version contains supplementary material available at 10.1186/s12943-022-01635-4.

## Background

Acute myeloid leukemia (AML) is a malignancy of hematopoietic stem cells or early progenitors resulting from the accumulation of genetic aberrations that disturb key biological processes. Mutations may occur in myeloid progenitor populations, which confer self-renewal capacity to the progenitors [1]. In the past decades, numerous AML associated gene alterations have been identified that can be broadly grouped into seven functional categories [2]. The most frequent category comprises mutations that activate signal transduction pathways and induce proliferation or survival of HSPCs, such as *FLT3*, *NRAS*/*KRAS* and *KIT*. A second category describes mutations or fusions in genes coding for transcription factors that are required for hematopoietic maturation, like *AML1-ETO* (*RUNX1-RUNX1T1*) and *CEBPA*. Mutations in epigenetic regulators like *IDH1/2*, *TET2*, *DNMT3A* and *ASXL1* comprise the third category of somatic events that are often acquired at an early stage [3]. The four remaining categories include aberrations in the Nucleophosmin (NPM1) gene, tumor suppressors and members of the spliceosome- and cohesin complexes.

Despite that current chemotherapies efficiently induce complete remission, AML patients frequently suffer from relapse and have low overall 5-years survival rates [4, 5]. Recurrence can emerge for example as a result of the expansion of pre-existing chemo-resistant subpopulations or by acquiring novel chemo-resistant subpopulations due to genomic alterations [6]. The advent of single-cell RNA sequencing provides revolutionary opportunities to assess the heterogeneity of cancer populations at the single-cell level and explore the transcriptional features of individual cell types, such as subpopulations contributing to the relapse. Several single-cell RNA sequencing studies revealed this transcriptional heterogeneity between and within tumor samples [7–9].

The prime aim of our study was to define the transcriptional changes between the Dx and Re samples. We used whole-exome sequencing (WES) to study the somatic mutations and copy number variations (CNVs) at Dx and Re. Furthermore, we applied single-cell RNA sequencing to analyze gene expression changes in AML samples between diagnosis and relapse. We profiled 5,612 high-quality cells at diagnosis and relapse from 6 AML patients, two intermediate risk cases with t(8;21) (*AML1-ETO*), three *DNMT3A* and one *NUP98/NSD1*. The latter four presented *FLT3*-ITD at the time of diagnosis and three of these patients were treated with the *FLT3* inhibitor Midostaurin. Our study provides novel insights into recurrence and unveils vulnerabilities that could serve as new entry points for targeting relapse AMLs.

## Methods

This is a brief description of the methods. A detailed description of each method is provided in Additional file 1.

### AML samples and cell preparation

We processed 6 paired Dx-Re bone marrow aspirates from adult AML patients, with *AML1-ETO* (*n* = 2 intermediate risk cases), *DNMT3A* (*n* = 3) and *NUP98-NSD1* fusion gene (*n* = 1). The latter four pairs presented *FLT3*-ITD at diagnosis with variable allele frequencies, and three of four patients were treated with *FLT3*-ITD inhibitor Midostaurin. Although *FLT3*-ITD is not an AML initiating lesion, nor an acknowledged World Health Organization (WHO) 2016 AML category, the mutation landscape and treatment is distinct from the *AML1-ETO* patients. Hence, we labelled patient s220 and s914 as “*AML1-ETO”* patients and termed patient s232, s292, s2275 and s3432 as “*FLT3*-ITD” patients. Patient characteristics are summarized in Supplemental Table 1. CD33/CD34 + cells were sorted into 384-well plates and stored at -80℃.

### Single cell SORT-seq

SORT-seq [10] is based on the integration of single cell FACS sorting (Fluorescence-Activated Cell Sorter) with the CEL-Seq2 protocol [11]. Single cell libraries were paired-end sequenced on an Illumina NextSeq500 at an average depth of ~ 30 M reads per library. After filtering out low quality cells (genes detected < 500 or UMI count > 12,000 or mitochondrial UMIs > 30% or ERCC reads > 20%), 5,612 high-quality cells were acquired and used for the following analyses (Supplementary Fig. 2A and Supplemental Table 1).

### Fusion genes detection

To quantify the reads per gene and detect fusion genes from bulk RNA-Seq, sequencing libraries were aligned to Gencode v37 reference genome version hg38 using STAR-Fusion v1.10.0 [12] in 2-pass mode, with parameters –*CPU 12 –FusionInspector validate –examine_coding_effect –denovo_reconstruct.*

### Whole-exome sequencing

WES libraries were generated as previously described [13]. Diagnosis and relapse samples were compared with samples collected at CR (Complete Remission). Somatic variant calling and CNV detection were performed with the Genome Analysis Toolkit (GATK).

### Pseudo-time trajectory analysis

We used Monocle3 [14] for pseudo-time analysis with default parameters, to assess the trajectories within the pairs. We used the DEGs obtained from Seurat 3.0 [15] to plot the dynamic changes of gene expression along the trajectories.

### Definition of leukemic stem cells and cycling genes

The 17-gene leukemic stem cell (LSC17) score was calculated based on the equation by Ng et al. [16]. Cell cycle phase scores were calculated using Seurat 3.0 function *CellCycleScoring* with default parameters.

## Results

### Whole exome- and gene fusion analyses reveal clonal aberrations

**Fig. 1:**
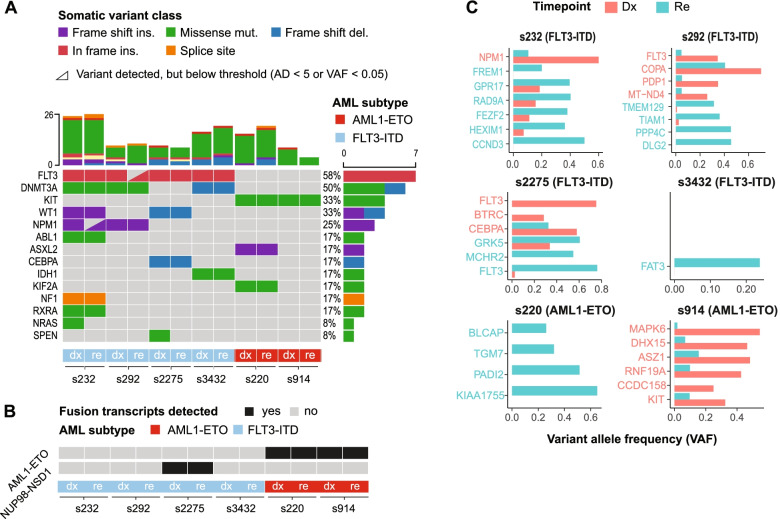
Whole exome- and gene fusion analysis between Dx and Re. (**A**) Oncoplot from WES showing 14 selected somatic mutations across 6 patients (red: *n* = 2 *AML1-ETO*; blue: *n* = 4 *FLT3*-ITD). We termed patient s232, s292, s2275 and s3432 as “*FLT3*-ITD”, although it is not an AML initiating lesion, nor an acknowledged WHO2016 AML category, but treatment with the FLT3 inhibitor Midostaurin is distinct from the *AML1-ETO* patients. Mutations with at least 5 reads on the ALT allele and VAF ≥ 0.05 are depicted as squares and the ones below this threshold are indicated as triangle. Vertical bars depict the number of mutations detected per sample; horizontal bars depict the (relative) frequency of a particular mutation. (**B**) Gene fusions detected from bulk RNA-seq. (**C**) Mutations with a VAF ≥ 0.2 at Dx or Re for which the VAF changed significantly. For all bars, *p* < 0.05, Fisher’s exact test with Benjamini–Hochberg correction. Red: mutations more abundant at Dx. Blue: mutations more abundant at Re

Clonal expansion and evolution is a major determinant of AML relapse [17]. To identify the genomic landscape at Dx and Re, we performed whole exome sequencing (WES) and gene fusion detection based on bulk RNA-sequencing. We detected between 4 and 26 somatic mutations per sample (Fig. 1A, Supplemental Table 2). This analysis confirmed the presence of an inframe insertion in the juxtamembrane domain (JMD) between amino acid 583 and 611 in all four *FLT3*-ITD patients as well as *AML1-ETO* fusion transcripts in the *AML1-ETO* patients (Fig. 1A, B, Supplemental Table 2). Other AML-associated somatic variants, such as *NPM1*, *WT1*, *CEBPA*, *IDH1*, *NRAS* and *DNMT3A* were detected for the *FLT3*-ITD patients, often in a patient-specific manner. For both *AML1-ETO* patients, the WES analysis revealed a *KIT* mutation that is associated with poorer prognosis and increased risk of relapse [18, 19].

Next, to identify clonal rearrangements that may have led to disease relapse, we screened for somatic mutations with a significantly altered variant allele frequency (VAF) between Dx and Re (VAF ≥ 0.2 and *p* < 0.05, Fisher’s exact test; methods; Fig. 1C). In the *FLT3*-ITD group, patient s232, WES and PCR analysis revealed two distinct *FLT3-*ITD mutations in the Dx sample, one of which one was lost at Re (*p* = 1.0 × 10^–3^; Fisher’s exact test based on WES reads; Fig. 1C, Supplemental Table 2). WES also revealed a *NPM1* mutation (type A^19^) at Dx, whose AF was decreased at Re (*p* = 8.2 × 10^–3^) and a low-abundant *NRAS* mutation at Dx (VAF = 0.087) that was undetected at Re (VAF = 0; *p* = 2.0 × 10^–4^; Fig. 1C). For patient s2275, WES showed considerably shorter ITDs at Re compared to Dx (*p* = 4.6 × 10^–41^), which were confirmed by PCR (Supplemental Table 3). This patient (s2275) had a *NUP98-NSD1* fusion at Dx and Re, consistent with the absence of *DNMT3A* and *NPM1* mutations [20, 21]. Finally, this patient displayed copy neutral loss of heterozygosity at the q-arm of chr13 (13q-LOH), which increases the allelic burden of the *FLT3*-ITD [22]. This aberration existed in a small fraction of Dx cells, but its abundance and that of the *FLT3*-ITD allele increased drastically at Re (Supplemental Fig. 1, Supplemental Tables 2 and 3). Patient s292 displayed *NPM1* and *DNMT3A* mutations that remained unaltered between DX and Re. Patient s3432 showed a retention of the *FLT3-*ITD, both in the insertion location and allelic ratio (WES and PCR). Somatic mutations in *FAT3* (VAF = 0.238, *p* = 4.3 × 10^–8^), *ITGB7* (VAF = 0.165, *p* = 1.32 × 10^–6^), *UBA2* (VAF = 0.117, *p* = 6.32 × 10^–3^) and *SLC4A3* (VAF = 0.135, *p* = 6.6 × 10^–3^) were significantly gained in the Re sample (Fig. 1C and Supplemental Table 2), whereas the last 53 Mb of chr7 was lost on one allele (Supplemental Fig. 1).

One of the *AML1-ETO* samples (s914) showed two distinct *KIT* mutations (VAF = 0.325; VAF = 0.138) at Dx, both of which were significantly reduced at Re (p < 4.7 × 10^–7^). Patient s220 gained a mutation in the *FAT3* gene as well as copy number aberrations at chr2 and chr15 at Re (Supplemental Fig. 1).

To summarize, we confirmed the presence of *FLT3-*ITDs and *AML1-ETO* in four and two patients respectively. Additional somatic aberrations in AML-associated genes were patient-specific. *FLT3-*ITDs were altered in two patients. In one patient, one of the two *FLT3-*ITDs was lost at Re. For patient s232, a *NPM1* mutation was detected at Dx (VAF = 0.6), but was decreased at Re (VAF = 0.1, *p* = 0.008, Fisher’s exact test). We also observed a significant reduction in two distinct *KIT* mutations in patient s914 between Dx and Re as well as patient-specific copy number changes at Re.

### Single cell transcriptomics reveals distinct AML-phenotypes at Dx and Re

Next, to better understand the transcriptional phenotypes, their differences and possible mechanisms that led to disease progression, we profiled bone marrow cells obtained at Dx and after Re using single cell transcriptomics. In brief, single CD33^+^ or CD34^+^ bone marrow cells were FACS-sorted into 384-well plates following the SORT-seq method [10], we acquired 5,612 single cell profiles, in which 4,129 unique transcripts from 1,678 genes were detected on average (Supplemental Fig. 2A, methods).

**Fig. 2:**
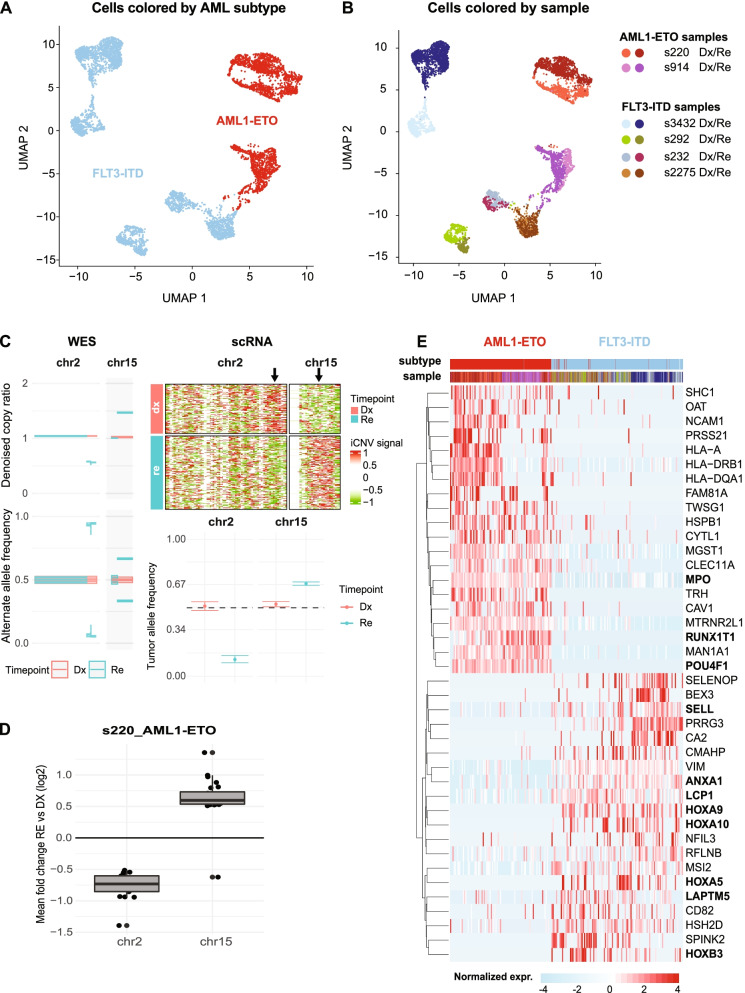
Single cell transcriptomics reveals distinct AML-phenotypes. (**A**) UMAP of the six AML pairs, colored by primary mutation (red: *AML1-ETO*; blue: *FLT3*-ITD); (**B**) UMAP colored by sample; (**C**) Copy number variation data derived from WES (left) and scRNA-seq (right) data for patient s220. Left: Relapse-specific copy number gain and loss at chr2 and chr15, respectively. Right: cell normalized gene expression signals (iCNV signal) in tiles of 3 Mb show the copy loss and gain at chr2 and chr15, respectively. The plot indicates that virtually all Re cells are affected, compared to none of the Dx cells. Bottom: tumor allele frequency at heterozygous SNPs confirms copy loss at chr2 and gain at chr15. (**D**) Boxplot of DEGs at the lost and gained segments of chr2 (*n* = 17 DEGs) and chr15 (*n* = 24 DEGs), respectively. (**E**) Heatmap showing the top 20 marker genes per primary mutation

After normalization, cells were clustered and visualized using the uniform manifold approximation and projection [23] (UMAP). *AML1-ETO* vs *FLT3-*ITD samples are separated by UMAP1 and Dx-Re pairs cluster relatively close together (Fig. 2A, B). Nevertheless, considerable heterogeneity between and within pairs exists (Fig. 2B). Strikingly, Dx-Re cells of *FLT3*-ITD patient s232 cluster in close proximity suggesting minor phenotypic and molecular alterations, even though this patient lost *NPM1* and *NRAS* mutation at Re. Similarly, the expression changes between Dx and Re cells of patient s914 were minor, despite the significant loss of two *KIT* mutations. In contrast, Dx cells of patient s3432 are completely separated from Re cells, although one mutation in the *FAT3* gene was detected in Re (VAF = 0.238) (Supplemental Table 2). Likewise, the Dx and Re cells of *AML1-ETO* patient s220 constitute distinct clusters, but only gained mutations in genes that are not associated with AML (Fig. 1C). Both patients did however display large copy number changes between Dx and Re (Supplemental Figs. 1 and 6A). Inferred CNV analysis confirmed that these aberrations were undetected for cells at Dx, but present for almost all cells at Re (Fig. 2C). As expected, these CNVs caused differential gene expression at these loci (Fig. 2D), such as *LOXL1* and *FAM81A* implicated in cancer progression [24].

Next, we looked for gene signatures that discriminated *AML1-ETO* or *FLT3-*ITD patients. These signatures include well-established *AML1-ETO* markers, like upregulation of the transcriptional co-repressor *RUNX1T1* (aka *ETO*), the transcription factor *POU4F1 *[25] and the myeloid differentiation protein *MPO *[26] (Fig. 2E, top). *FLT3*-ITD samples on the other hand are characterized by induced expression of *VIM*, *ANXA1*, *MSI2* and *LAPTM5*. Other genes tend to be overexpressed only in a subset of the samples: *HLA* genes are overexpressed in *AML1-ETO* patient s220, but not in s914. In the *FLT3-*ITD samples, *HOXA5* and *HOXB3* genes that are overexpressed in *NPM1*-mutated AML [27], appear overexpressed in a patient-specific manner (Fig. 2E, bottom). Closer inspection of these and other *NPM1*-marker genes showed that these genes are indeed significantly higher expressed in the *FLT3*-ITD samples with an additional *NPM1* mutation (*NPM1*^mut^) compared to *NPM1*^WT^ samples (FC > 1.5 and p < 6.0 × 10^–15^; Supplemental Fig. 2D). Notably, *HOX*-genes are also highly expressed in *FLT3-*ITD patient s2275. In these samples, we detected a *NUP98-NSD1* fusion gene that is characterized by upregulation of *HOXA* and *HOXB* genes [28] (Supplemental Fig. 2D).

In summary, single cell transcriptomics showed distinct clustering of *AML1-ETO* vs *FLT3-*ITD patients. Differential analysis confirmed upregulation of well-established marker genes as well as elevated expression of *HOX* genes in *NPM1*^mut^ and the *NUP98-NSD1* positive *FLT3-*ITD samples. On a global level, the transcriptional changes between Dx and Re are poorly explained by SNPs and INDELs in AML-associated genes, but rather seem to be associated with large-scale CNVs. To gain a deeper understanding of the mechanisms underlying these changes, we subsequently performed an in-depth analysis of Dx-Re pairs per AML-subtype and in a patient-specific setting.

### Dx-Re transcriptomic changes are patient specific

**Fig. 3:**
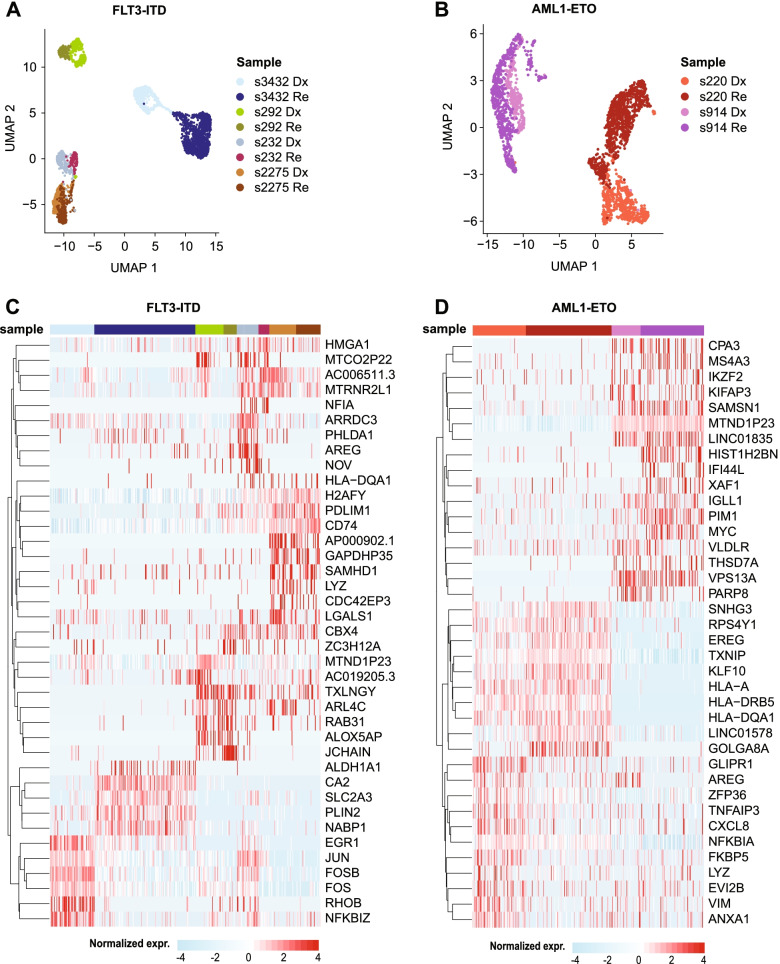
Single cell transcriptomics reveals heterogeneity amongst patients. (**A**) UMAP of the four sample pairs with a *FLT3*-ITD, colored by sample (red: Dx; blue: Re); (**B**) UMAP of the two *AML1-ETO* sample pairs, colored by sample; (**C**) Heatmap displaying the top 5 marker genes per sample (*FLT3*-ITD); (**D**) Heatmap displaying the top 10 marker genes per sample

Given this high intra- and inter-patient heterogeneity, we focused on the Dx-Re differences per patient in the remainder of this study. For this, we separated the UMAPs of the *FLT3*-ITD and *AML1-ETO* patients (Fig. 3A, B) and computed the differentially expressed genes between the Dx-Re pairs per patient. This analysis reinforced the notion that the differences in transcription between Dx and Re are highly patient-specific (Supplemental Figs. 2B, C, 3C, D).

The *FLT3*-ITD patients show a modest separation between the Dx and Re samples of patient s232 (Fig. 3A). Cluster analysis revealed two clusters at diagnosis (cluster 1–2) and one at relapse (cluster 3, Supplemental Fig. 3A). Re cells lost expression of members of the *AP-1* transcription factor, like *FOS*, *FOSB* and *ATF3* that were highly expressed in Dx cluster 1 (Supplemental Fig. 3B). Gene ontology (GO) analysis confirmed significant loss of expression for these and other genes involved in *AP-1*/*ATF-2* related transcription at Re (Supplemental Fig. 3C). Furthermore, we evaluated the expression level of genes involved in PI3K/AKT/mTORC pathway, in which mTORC1 controls ribosomal biogenesis and protein translation. We found the targets of mTORC1, like *RPS6KB1* and *EIF4E*, were differentially expressed at Re (Supplemental Fig. 3D), suggesting a pathway shift from AP-1 to mTORC1. Besides, we observed the upregulation of the upstream *K/NRAS* genes in Re, which may be markers for diagnosis/prognosis and treatment targets.

The UMAP for patient s292 showed 3 distinct clusters (Supplemental Fig. 4A). DEG between Dx clusters 1 and 2 revealed *IDH1*, an enzyme in the TCA cycle, and *RAB31* involved in membrane fusion and exocytosis in clusters 1, whereas *MPO* and *PROM1*, markers for GMP cells, are differentially expressed in cluster2 (Supplemental Fig. 4B, C). Cells in cluster 3 originate from the Re sample and overexpressed genes like *DDIT4 *[29], *PIM3 *[30] and *CD74 *[31] were previously associated with poor prognosis (Supplemental Fig. 4B). GO analysis indicated regulation of cell death and apoptotic process terms in cluster 3 (Supplemental Fig. 4C).

The copy-neutral LOH at chr13q could be detected from the alternate allele frequency in the WES and scRNA-seq data of patient s2275 (Supplemental Fig. 5A). However, no genes located on chr13q were differentially expressed at Re compared to Dx, with the exception of *ELF1* (twofold downregulated at Re). Single cell expression analysis revealed 5 clusters. Cluster 1 mainly originated from Dx cells, whereas cluster 5 almost entirely consisted of Re cells. Clusters 2–4 however were a mixture between Dx and Re cells (Supplemental Fig. 5B, C). DEGs revealed few differences between cluster 1 and 5, such as *RNU4ATAC* and *RYBP* involved in RNA biosynthesis and metabolism that are differential expressed in cluster 1 (Dx) (Supplemental Fig. 5C, D), whereas *ITM2A* and *CLEC12A* for leukocyte activation and *LDHA* for ribonucleotide metabolism are differentially expressed in cluster 5 (Re) (Supplemental Fig. 5C, D). The minor differences between Dx and Re is consistent with the fact that AML-associated mutations, such as *FLT3*-ITD, *WT1*, *CEBPA* and *NUP98-NSD1* were present at Dx and retained at Re (Fig. 1A, B, Supplemental Table 2).

**Fig. 4:**
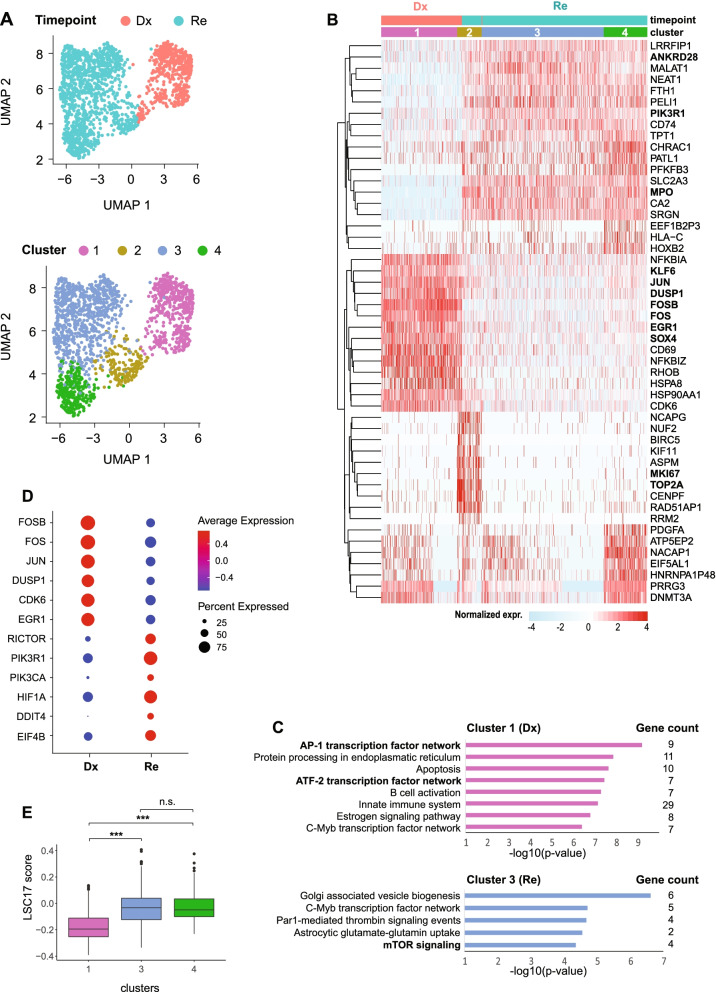
Pathway switch between AP-1 and RAS signaling in high risk *FLT3*-ITD (s3432). (**A**) UMAP of Dx and Re cells for *FLT3*-ITD patient s3432 colored by timepoint (top) or cell cluster (bottom). (**B**) Heatmap displaying the top 10 cluster marker genes. Color represents row normalized expression values. (**C**) Overrepresented GO terms (category: biological pathway) in cluster 1 (Dx) and 3 (Re). *P*-values: hypergeometric test (BH-corrected). (**D**) The expression of genes related to AP-1 transcription factor network and RAS signaling pathway in each timepoint. (**E**) Calculation of LSC17 score for each cluster, and *p*-value was calculated using Student’s t-test. * *p* < 0.05, ** *p* < 0.01, *** *p* < 0.001

The Dx and Re cells of patient s3432 formed distinct clusters that are highly separated from each other and the other *FLT3*-ITD patients (Fig. 3A). Cluster analysis detected four groups of cells that largely separated Dx (cluster 1) from Re cells (cluster 2–4; Fig. 4A). Cluster 1 had a characteristic gene signature of transcription factors involved in proliferation and cell growth (e.g., *JUN*, *FOS*, *FOSB*, *EGR1*, *SOX4* and *KLF6*) that were significantly downregulated in the Re clusters (Fig. 4B, C). The Re-specific clusters 3–4 upregulated genes involved in the RAS/mTORC pathway, such as *ANKRD28* and *PIK3R1*, whereas cluster 2 is hallmarked by cell cycle related genes, such as *TOP2A* and *MKI67*. Pathway enrichment analysis confirmed the overrepresentation of AP-1/ATF2 transcription factors in cluster 1 (Dx) and additionally revealed upregulation of genes involved in mTOR signaling, like *RICTOR*, *PIK3R1* and *HIF1A* in cluster 3 (Re; Fig. 4C, D). This suggests a pathway switch from AP-1 in the diagnosis cells towards mTOR in the relapse cells. We further observed that *KRAS* and *NRAS*, genes upstream of mTORC, were also overexpressed in the Re sample (Supplemental Fig. 6C). Interestingly, cluster 4 in relapse is characterized by elevated exocytosis (Supplemental Fig. 6D) and increased expression of genes related to Tim-3-galectin-9 Secretory Pathway (e.g. *ADGRL1*, *HAVCR2* and *LGALS9*) that protect AML cells against from the host immune system in an mTOR dependent manner [32] (Supplemental Fig. 6E), in particular from NK- and T-cell action. Finally, the leukemia stem cell (LSC) score, a 17-gene signature (LSC17) that correlates with aggressiveness of the leukemia and a poor outcome [16] was significantly higher in the Re clusters 3 and 4 compared to the Dx cluster 1 (Fig. 4E). For the other *FLT3*-ITD patients, none of the clusters had an elevated LSC17 score (data not shown).

**Fig. 5:**
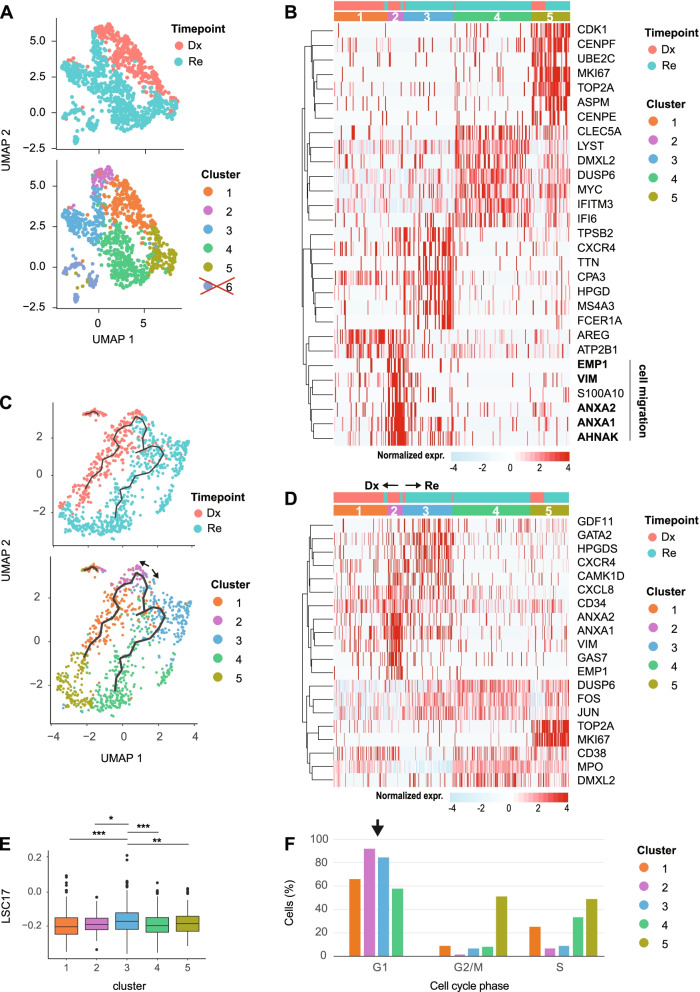
Putative LSCs detected in *AML1-ETO* pair (s914). (**A**) UMAP of Dx and Re cells for *AML1-ETO* patient s914, colored by timepoint (top) and cell cluster (bottom). Cells in cluster 6 express ambiguous marker genes, and may be doublets or contaminated by ambient RNA and were discarded (see also Supplemental Fig. 6). (**B**) Heatmap depicting the top 7 cluster markers. Color represents row normalized expression values. (**C**) Pseudo-time trajectory colored by timepoint (top) or cell cluster (bottom). (**D**) Heatmap showing representative genes per cluster. (**E**) LSC17 score per cluster. * *p* < 0.05, ** *p* < 0.01, *** *p* < 0.001, Student’s t-test. (**F**) Barplots depicting the relative cell abundance per cell cycle phase (inferred from marker gene expression) for each cell cluster. Arrow: cells in cluster 2 and 3 predominantly reside in the G1 phase

### Leukemic stem cell-like cells in *AML1-ETO*

Higher *MPO*, a marker for granulocyte/monocyte progenitors (GMPs) expression [26] within both the *AML1-ETO* patients (Fig. 2E) implies that most cells are arrested at a “GMP-like” stage. The Dx and Re cells of AML1-ETO patient s220 were more separated compared to those of patient s914 (Fig. 3B, Supplemental Fig. 2C). 41 of the DEGs are transcribed at the amplified or lost loci and their altered transcription may in turn deregulate other genes.

Analysis on Dx-Re showed that the number of DEGs shared between these two *AML1-ETO* patients is minimal as for the *FLT3-*ITDs (Supplemental Fig. 2C). Therefore, we performed an in-depth analysis on the transcriptional dynamics between Dx and Re separately for these two patients. Focusing on patient s914 first, the synergic oncogenes (*PIM1* and *MYC *[33]) responsible for tumorigenesis were co-differentially expressed at Re compared to Dx. Cluster analysis revealed five groups of cells (Fig. 5A, B) and a small cluster of scattered cells that expressed signatures of progenitors (*CD34*), erythrocytes (*HBB*), monocytes (*LYZ*), B-cells (*MSA41*) and cell cycle related genes (*TOP2A*, *MKI67*) (Supplemental Fig. 7) likely resulting from ambient RNA or cell doublets and hence were discarded in subsequent analyses.

Cluster 1 mainly consist of Dx cells and differentially expressed genes for differentiation and resistance to apoptosis, like *AREG *[34]. Interestingly, cells in cluster 2 express *CD34* as well as genes involved in cell migration (*ANXA1 *[35], *ANXA2 *[36], *VIM *[37] and *EMP1 *[38]) but lacked the expression of *MPO* (Fig. 5B, D). To investigate whether and from which Dx cluster these potential Re LSCs originate, we aligned cells in pseudo-time based on the gradient of transcriptional differences using Monocle3. This trajectory analysis suggested a continuous transition between the Dx and Re sample (Fig. 5C). Cells in cluster 2 and 3 differentially expressed genes for hematopoietic stem cell maintenance (*GDF11 *[39], *GATA2 *[40]) and differentiation (*GAS7 *[41], *CAMK1D *[42]) markers as well as *CD34* (Fig. 5B, D), indicating cluster 2 and 3 are the putative starting points of this trajectory. Besides, cluster 2 and 3 overexpressed genes *CXCR4 *[43] and *CXCL8 *[44] for tumor microenvironment (Fig. 5B, D). In line with those findings, we calculated the LSC17- and cell cycle scores for all clusters. We observed that cells in Dx cluster 3 have the highest LSC17 score followed by Re cluster 2 (Fig. 5E). Moreover, cells from cluster 2 and 3 mainly reside in the G1 phase of the cell cycle (Fig. 5F). Interestingly, the trajectory suggest that these cells differentiate into a population of cells that display *DUSP6* and AP-1 related genes like *JUN* and *FOS* in the Re-specific clusters 3 and 4 (Fig. 5D).

**Fig. 6:**
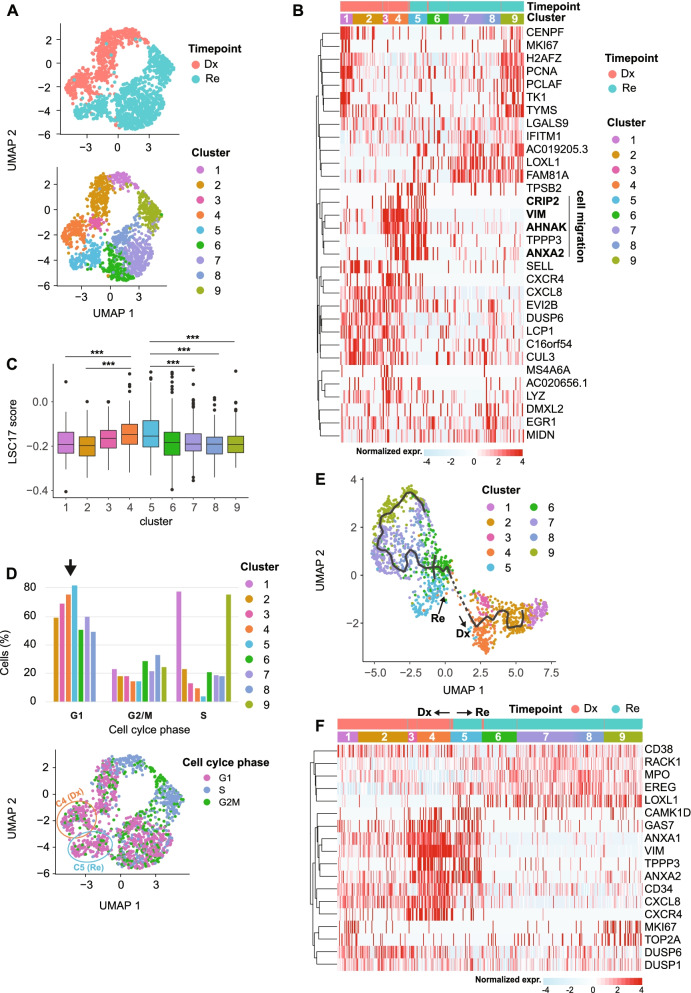
Putative LSCs detected in *AML1-ETO* pair (s220). (**A**) UMAP of Dx and Re cells for *AML1-ETO* patient s220, colored by timepoint (top) and cell cluster (bottom). (**B**) Heatmap depicting the top 5 marker genes per cluster. Color represents row normalized expression values. (**C**) LSC17 scores per cluster. * *p* < 0.05, ** *p* < 0.01, *** *p* < 0.001, Student’s t-test. (**D**) top: Barplots depicting the relative cell abundance per cell cycle phase (inferred from marker gene expression) for each cell cluster. Arrow: cells in cluster 4 and 5 predominantly reside in the G1 phase. Bottom: UMAP colored by cell cycle phase. (**E**) Pseudo-time trajectory colored by cell cluster (**F**) Heatmap depicting representative marker genes per cluster/inferred timepoint

UMAP shows that s220 cells separate according to Dx and Re which partitioned into 9 clusters (Fig. 6A). Clusters 1–4 contained Dx cells that were enriched for *CXCL8* and *CXCR4*, genes associated with the interaction between leukemia blasts and stromal cells [43, 44]. Clusters 5–9 exclusively contained Re cells and were marked by expression of *LOXL1* and *FAM81A* (Fig. 6B). Cell cycle-related genes (*MCM6*, *TOP2A*, *MKI67*) were highly expressed in cluster 1 and 9. Cluster 4 (Dx) and 5 (Re) are in close proximity to each other and share marker genes, such as *CAMK1D*, *GAS7*, *ANXA1*/*2*, *VIM* and *CD34* (Fig. 6B, Supplemental Fig. 8A) suggesting that they are LSCs.

### Alternative “branching” from Re and Dx LSC-like cells in *AML1-ETO*

Given the high similarities between clusters 4 and 5 and their elevated *CD34* expression, we hypothesized that these clusters might be enriched in LSCs. Analysis showed that these clusters indeed have the highest LSC17-score and contain cells that reside predominantly in the G1 cell cycle phase (Fig. 6C, D). To better understand the transcriptional dynamics of cell populations originating from these LSCs, we applied pseudo-time gene expression analysis (Fig. 6E). This analysis reveals a trajectory starting from the presumed LSCs cluster 4 and 5 towards more differentiated cells that predominantly reside in the S-phase of the cell cycle and exhibit elevated expression of genes like *TOP2A* and *MKI67* (Fig. 6D-F). For the Dx branch, genes involved in self-renewal that impede differentiation (*GAS7* and *CAMK1D*) or are associated with cell migration (*TPPP3*, *VIM*, *ANXA1*/*2*) had elevated expression in cluster 4. We hypothesized that all other clusters of cells originate from this presumed Dx LSC population. Indeed, we observed a downregulation of these markers when cells are traced along the trajectory from cluster 4 to cluster 1 which is consistent with their differentiation into more mature myeloid cells. Furthermore, *DUSP1* and *DUSP6*, genes required for cell differentiation and proliferation were upregulated as cells ‘moving away’ from cluster 4 along the Dx branch (Fig. 6F). In the Re branch, *TPPP3*, *VIM*, *ANXA1*/*2*, *GAS7* and *CAMK1D* were upregulated in cluster 5 to a similar extent as in cluster 4. Compared to the more gradual downregulation in the Dx branch, these markers were largely lost when cells “branched” from cluster 5 to cluster 6 (Fig. 6F**)**. The Re trajectory (cluster 5 towards cluster 9) is hallmarked by upregulation of numerous genes required for differentiation, leukemia progression and chemo-resistance, including *RACK1 *[45], *EREG *[46] and *LOXL1 *[24] (Fig. 6F). Another striking difference between the Dx and Re is that genes associated with the tumor microenvironment, the interaction between stroma cells and leukemic blasts (*CXCR4* and *CXCL8*) were lower expressed in cluster 5 (Re) compared to cluster 4 (Dx, Fig. 6B). Gene Ontology analysis further revealed up-regulated genes in Dx enriched with terms associated with immune- and inflammatory response, whereas translation and biosynthesis related processes were highly enriched in Re (Supplemental Fig. 8B).

In summary, our data reveals a heterogeneous mixture of cells in the *AML1-ETO* patients. Dx vs Re cells were more heterogeneous in patient s220 compared to s914. Part of this heterogeneity is caused by large-scale CNVs that affect transcription at the affected loci. Deregulation of these genes may affect other genes and thus increase the transcriptional differences between Dx and Re. Interestingly in both patients, we found cells with a significantly elevated LSC17-score that are predominantly in the G1-phase. These cells appear to be at the origin of other cell populations that develop/branch in a way that is sample and stage specific. The signature genes for LSCs might be potentially therapeutic targets to improve the efficiency of AML treatment.

## Discussion

To gain insight into the heterogeneity between AML subtypes and within Dx-Re pairs, we profiled the exome, gene fusions and single cell transcriptome of four *FLT3-*ITD and two *AML1-ETO* Dx-Re sample pairs. Here, we focussed on CD33/CD34 + stem- and progenitor cells using plate-based single cell technology. Clustering and differential expression analysis of single cell transcriptomes showed extensive intra- and inter-blasts heterogeneity. The strongest transcriptional differences were associated with patient-specific large scale copy number variation. Another source of heterogeneity were somatic variants (SNPs and INDELs) that point to highly patient-specific abundance and dynamics of AML clones.

We were unable to identify a common molecular mechanism that caused AML relapse across all patients. Instead, our data showed highly heterogeneous genomes and transcriptomes that were patient- and even disease stage specific. The question to what extend relapse-inducing molecular mechanisms are truly patient-specfic, or can be grouped into certain classes, calls for a much larger AML patient cohort than we currently profiled. Despite this limitation, we found differences in underlying resistance mechanisms that are not exclusively caused by clonal rearrangements. One patient showed a pathway switch from AP-1 dependency at Dx to mTOR signaling at Re that appeared to be independent of altered somatic mutations, suggesting that clonal rearrangements are not causing the relapse in this patient [47]. In contrast, significantly altered mutations (e.g., decrease/loss of *NPM1* and *KIT*) in other patients were accompanied by minor transcriptional differences. Furthermore, the presence of quiescent LSCs that escape conventional therapeutic interventions could explain recurrence in the absence of clonal rearrangements [8, 48, 49]. In agreement with this hypothesis, we detected transcriptionally similar LSC-like cells in the Dx and Re samples of the two otherwise distinct *AML1-ETO* samples. While the expression of these LSC populations is similar at Dx and Re, their differentiation trajectories are remarkably different.

Our results share and extend findings from other recent single cell AML studies. One of them used a more stringent sorting approach to profile the transcriptome of leukemia initiating cells and reported increased BCL2 and CXCR4 signaling in relapse [8]. These genes were also highly expressed in some, but not all, of our diagnosis and relapse samples and also not consistentlty induced at relapse. Other studies based on larger numbers of unsorted cells [26] or deconvolution of bulk RNA-sequencing [50] detected a hierarchy from primitive to differentiated AML tumor cells. They showed that the composition of tumor cells is patient-specific and associated with chemotherapy and drug sensitivity. Although our sorting strategy removed the more mature blasts, we still found strong sample-to-sample heterogeneity within the CD33/CD34 + stem- and progenitor cells. Furthermore, we found sample-specifc activation of pathways such as mTOR and RAS signaling.

Taken together, our study and others have provided a first step to unravel the highly complex nature of the AML bone marrow using single cell technology. It is evident that a much better understanding is needed to ultimately reduce relapse rates and improve long-term survival. Rapid advances in single cell technology now allow profiling of the genome, transcriptome and epigenome for thousands of cells in parallel. This multi-layered information is needed to identify and trace distinct clones, their phenotypes and their (in)sensitivity to therapeutic interventions.

## Conclusions

In conclusion, this study indicates that samples with extensive copy number variations showed larger transcriptional differences between Dx and Re, compared with those with SNPs and INDELs only. Importantly, we found pathway switches (e.g., *AP-1* to *mTOR*) with few differential somatic mutations and vice versa. In two pairs of *AML1-ETO* samples, we found leukemic stem cell-like cells that shared the expression of common characteristic genes.

## Supplementary Information

**Additional file 1**: Supplemental methods. 

**Additional file 2: Supplemental Table 1. **Clinical information and sequencing details of the patients. 

**Additional file 3: Supplemental Table 2. **Dynamic changes of mutations between Dx and Re (WES) and detected fusion genes from bulk RNA-Seq. 

**Additional file 4: Supplemental Table 3. **Characterization of *FLT3*-ITD at diagnosis and relapse. 

**Additional file 5:** **Supplemental Figure 1.** Copy number variation based on WES data.Denoised copy ratios and alternate allele frequencies for each sample at Dx (red) and Re (cyan).

**Additional file 6: Supplemental Figure 2. **Quality Control of single cell data. (A)Violin plots depicting the detected number of genes (top) and unique transcripts (bottom) per cell. (B) Venn diagram showing the number of DEGs between the Dx and Re sample, for a pairwise comparison in the four *FLT3*-ITD patients. Very few DEGs are shared between patients.  (C) Same as (B), for the two *AML1-ETO *patients.  (D) Violin plots depicting gene expression at known NPM1 target genes in *FLT3*-ITD samples with- and without *NPM1* mutation. 

**Additional file 7: Supplemental Figure 3. **Single cell landscape of Single cell landscape of *FLT3*-ITD patient s232. (A) UMAP of Dx and Re cells for *FLT3*-ITD patient s232 colored by timepoint (top) or cell cluster (bottom). (B)Heatmap displaying the top 20 cluster marker genes. Color represents row normalized expression values. (C) Overrepresented GO terms (category:biological pathway) in cluster 1 (Dx) and 3 (Re). *P*-values: hypergeometric test(BH-corrected). (D) Gene expression of selected mTORC1 pathway members. 

**Additional file 8: Supplemental Figure 4. **Single cell landscape of *FLT3*-ITD patient s292. (A) UMAP of Dx and Re cells for *FLT3*-ITD patient s292 colored by timepoint (top) or cell cluster (bottom). (B) Heatmap displaying thetop 20 cluster marker genes. Color represents row normalized expression values.(C) overrepresented GO terms (category: biological pathway) per cluster. 

**Additional file 9: Supplemental Figure 5. **Single cell landscape of *FLT3*-ITD patient s2275. (A) Copy number variation data derived from  WES (left) and scRNA-seq (right) data for patient s2275. Left: Copy neutral loss of heterozygosity of q arm of chr13. Right: cell normalized gene expression signal (iCNV signal). (B) UMAP of Dx and Re cells for *FLT3*-ITD patient s2275 colored by timepoint (top) or cell cluster (bottom). (C) Heatmap displaying the top 20 cluster marker genes. Color represents row normalized expression values. (D) overrepresented GO terms (category: biological pathway) for clusters 1 (Dx) and 5 (Re). 

**Additional file 10: Supplemental Figure 6. **Relapse cells of *FLT3*-ITD patient s3432 are associated with exocytosis. (A) Copy number variation data derived from WES (left) and scRNA-seq (right) data for patient s3432. Left: Relapse-specific copy number loss at chr7. Right: cell normalized gene expression signal (iCNV signal). (B) Boxplot of DEGs at the lost segments of chr7 (*n*=8 DEGs). (C) Gene expression of selected RAS-pathway members. (D) overrepresented GO terms (category: biological pathway) for clusters 4. (E) Selected genes associated with exocytosis. Color depicts relative expression; size depicts the relative number of cells for which at least one transcript was detected. ** *p* < 0.01, *** *p* < 0.001,Wilcox rank sum / Mann-Whitney U test. 

**Additional file 11: Supplemental Figure 7. **Cells in cluster 6 (blue circle, s914 *AML1-ETO*) simultaneously express hematopoietic stem-/progenitor- (*CD34*), monocyte (*LYZ*), B-cell (*MS4A1*), erythrocyte (*HBB*) and cell cycle (*TOP2A, MKI67*) marker genes. This indicates that these cells are doublets or contaminated by ambient RNA and were discarded from further analysis. Color bar represents the expression level of corresponding genes. 

**Additional file 12: Supplemental Figure 8. **Single cell landscape of *AML1-ETO *patient s220. (A) Heatmap displaying the top 20 cluster marker genes. Color represents row normalized expression values. Marker genes shared between cluster 4 (Dx) and 5 (Re) are highlighted inside a black rectangle. (B) overrepresented GO terms (category: biological pathway) at Dx and Re. 

## Data Availability

The high-throughput datasets have been deposited in the European Genome-phenome Archive. The accession numbers for single cell RNA-seq, bulk RNA-Seq and Whole exome sequencing datasets are EGAD00001008373, EGAD00001008374 and EGAD00001008375, respectively.

## Abbreviations

AML: Acute Myeloid Leukemia
HSPCs: Hematopoietic stem or progenitor cells
Dx: Diagnosis
Re: Relapse
CR: Complete remission
WES: Whole-exome sequencing
CNVs: Copy number variations
FACS: Fluorescence-Activated Cell Sorter
WHO: World Health Organization
GATK: Genome Analysis Toolkit
LSC17: 17-Gene leukemic stem cell
JMD: Juxtamembrane domain
ITD: Internal tandem duplication
UMAP: Uniform manifold approximation and projection
GMPs: Granulocyte/Monocyte progenitors
