# Supplementary material for: Longitudinal single-cell transcriptomics reveals distinct patterns of recurrence in acute myeloid leukemia
Source: Mol Cancer. 2022 Aug 19;21:166. doi: 10.1186/s12943-022-01635-4 (PMC9389773; doi:10.1186/s12943-022-01635-4)
Supplement: Supplementary file 1 — Additional file 1: Supplemental methods. [file 12943_2022_1635_MOESM1_ESM.pdf]

Supplemental Methods

AML samples

In total, 6 paired Dx-Re bone marrow aspirates from adult AML patients, aged between 31 and 69 and diagnosed with *AML1-ETO* (n=2 low risk cases) or *FLT3*-ITD (n=1 intermediate and n=3 high risk) were analyzed from the AMLSG BiO Registry study (NTC 01252485). The phenotypic characterization (*AML1-ETO* and *FLT3*-ITD) was performed at the reference laboratory of the German and Austrian AML Study Group (AMLSG). All patients received intensive standard induction chemotherapy with cytarabine and an anthracycline (7+3 regimen) followed by high-dose cytarabine consolidation cycles. Patient characteristics are summarized in **Supplementary Table S1**. Informed consent for both treatment and biobanking of leukemia samples according to the Declaration of Helsinki was given by all patients. Approval was obtained from the ethical review board of the University of Ulm (Ethikkommission der Universität Ulm).

Cell preparation

Bone marrow aspirates from AML patients were processed using density gradient centrifugation to isolate mononuclear cells, viably frozen in medium containing 10% DMSO and shipped with dry ice. Cryopreserved AML samples were thawed at 37℃. After washing with cold PBS, cells were resuspended in 500µl PBS with 0.2% human serum and incubated for 10 minutes on ice. After centrifugation and removing the supernatant, cells were resuspended in 200µl cold PBS with 20µl PE-conjugated CD33 and 20µl APC-conjugated CD34 (eBioscience). Cells were stained for 30 minutes on ice, washed with cold PBS with 0.5% BSA and resuspended in 500µl PBS with 0.5% BSA and 1µl 7-AAD (eBioscience). After incubation for 10 minutes, CD33/CD34+ cells were sorted into 384-well plate (BioRad) containing well-specific primers (100nl, 0.75pmol/µl) and 5µl mineral oil (Sigma-Aldrich) on the BD FACS Aria cell sorter. After sorting plates were sealed and span down for 2 minutes at 2000g, snap frozen on dry ice and stored at -80℃ until use.

Single cell SORT-seq

We applied SORT-seq[1], a method that integrates single cell FACS sorting (Fluorescence-Activated Cell Sorter) with the CEL-Seq2 protocol[2] to measure gene expression at single cell resolution. External RNA Controls Consortium (ERCC) transcripts were spiked-in to detect empty wells, low quality mRNA and failed reactions. The frozen plates were centrifuged at 1400 RPM for 2 minutes at 4°C before processing. Processing of single cell plates included first strand synthesis and barcoding in the 384 well plate. mRNA from the single cells were pooled and batch amplified by in vitro transcription (IVT) (Invitrogen # AM1334). Sequence libraries were prepared with Phusion High-Fidelity Polymerase (NEB).

Bulk RNA-seq

Total RNA was extracted using Quick-RNA Microprep Kit (Zymo Research) according to the manufacturer protocol with DNaseI treatment. The RNA concentration was quantified with Qubit Fluorometer (Invitrogen). RNA libraries were prepared with KAPA RNA HyperPrep Kit with RiboErase (HMR) kit (Roche) following the manufacturer's recommendations. RNA-seq libraries were paired-end sequenced on an Illumina Nextseq 500 at an average depth of ~30M reads.

Detection of *FLT3*-ITD at diagnosis and relapse

The presence of *FLT3*-ITD was detected by DNA-based PCR followed by capillary electrophoresis. The detailed procedures were described previously[3].

Data analysis

Sequencing and mapping

Single cell libraries were pair-end sequenced on an Illumina NextSeq500 at an average depth of ~30M reads per library and demultiplexed using bcl2fastq version 2.15.0.4 with default settings. We used STAR version 2.7.2b[4] to map the 42nt long read1 to human reference genome hg38. Next, we used UMI-tools[5] to reconstruct the gene by cell UMI count matrix from the BAM file.

Normalization, dimensionality reduction and cluster analysis

We used the Seurat v3[6] R-package for downstream analysis. First, low quality cells (genes detected < 500 or UMI count > 12,000 or mitochondrial UMIs > 30% or ERCC reads > 20%) were discarded (**Supplementary Figure 2A)**. Ribosomal and mitochondrial genes were also discarded prior to normalization. Cells from all libraries were concatenated and $\log_2$ normalized. Next, we applied principal component analysis (PCA) on the 2,000 most variable genes to reduce the dimensionality of the dataset and retained the 50 components for cluster analysis and the identification of marker genes. Cluster analysis was run using the Louvain algorithm. Cluster markers were identified using the Seurat function *FindAllMarkers* with parameters *min.pct*=0.25, *logfc.threshold*=0.5 and *only.pos*=FALSE. Marker genes discriminating two clusters or Dx from Re cells were obtained usng the Seurat function *FindMarkers* with parameters *min.pct*=0.25, *logfc.threshold*=0.5, *min.diff.pct*=0.2 and *only.pos*=FALSE. All marker genes with adjusted p-value > 0.01 were discarded.

Whole exome sequencing analysis

We used the GATK toolkit version v4.2.0[7] to detect short somatic variants following the GATK best practices workflows "*Data pre-processing for variant discovery*" and "*Somatic short variant discovery (SNVs + Indels)"* with small modifications. Briefly, paired-end reads were aligned using BWA version 2.2.1[8], discarding reads with MAPQ < 20. PCR duplicates were marked using Sambamba 0.8.0 *markdup*[9] and base quality scores were recalibrated using the GATK functions *BaseRecalibrator* and *ApplyBQSR* . Variants were called using the *panel of normals* (PON) and *gnomAD* VCF file provided in the GATK resource bundle in two modes: Dx and Re as tumor samples and Cr as a germline control, or all three samples as tumor only. The rationale for this approach is that variants present (at low frequency) in the Cr sample (due to minimal residual disease) are sometimes discarded as germline. Variants were filtered with *FilterMutectCalls* and annotated with the Ensembl Variant Effect Predictor (VEP) version 104[10].

Variants were discarded when ANY of the following conditions were satisfied:

- The variant FILTER status was unequal to "PASS" or "slippage."
- The variant had less than 5 reads on the alternative allele (AD < 5) in the Dx, Re and Cr samples;
- The variant allele frequency was below 0.05 (VAF < 0.05) in the Dx, Re and Cr samples;
- The variant had a gnomAD allele frequency $\geqslant 1.0 \times 10^{-3}$;
- The variant allele frequency did not change significantly between Dx vs CR or Re vs CR (p-adjusted $\geqslant$ 0.01; Fisher's exact test)
- The variant allele frequency at Cr exceeded 0.2 ($VAF_{CR} > 0.2$)

Mutations were visualized using the *maftools* R-package[11] and listed in supplemental table 2.

Somatic copy number variation analysis

Somatic CNV were detected from Dx and Re whole exome libraries using GATK v4.2.0, following the GATK "*Somatic copy number variant discovery"* workflow. In brief, a CNV panel of normal (PON) was constructed from the complete remission (CR) samples and CNVs were called from the Dx and Re samples, using CR samples as patient-matched normal samples. Called CNV segments were used as input for inferred copy number analysis (iCNV) using single cell RNA-seq data (described below).

Inferred copy number variation analysis

Copy number variation was inferred by tiling the genome into 3 Mb windows. Gene expression counts for genes overlapping each window where summed. Windows were log normalized followed by Z-scoring of cells (mean=0, sd=1) and smoothing using a running median with k=3 windows. Data was visualized using the *ComplexHeatmap* R-package[12].

To detect CNVs from altered allele frequencies at single cell resolution, we first modified the BAM files and prefixed each cell barcode with a sample specific pseudo-barcode. Next, Dx and Re BAM files were merged and heterozygous positions were detected using the program *BAFExtract*[13] and converted to VCF. This VCF was used to count UMIs supporting the REF or ALT allele at single cell level using CellSNP-lite[14]. To discard possible sequencing artifacts, somatic mutations and low coverage SNPS, we computed the binomial confidence interval (α=0.05) from the total REF and ALT counts at Dx; the timepoint that did not show the considered copy number aberrations. SNPs with a total depth < 20 UMIs or a binomial confidence interval that did not contain the expected AF=0.5 were discarded. Because the SNP counts are low on a single cell level, we aggregated all SNP counts inside a given CNV segment for each cell to increase statistical confidence. To this end, we first determined at each SNP whether it supported the tumor allele as the REF or ALT using the aggregated UMI counts from Dx and Re. UMIs supporting the tumor allele (ALT or REF) were subsequently summed over all heterozygous positions per cell and divided by the total UMI depth (REF + ALT) to obtain a “tumor allele frequency” per cell.

References

1. Muraro MJ, Dharmadhikari G, Grün D, et al. A Single-Cell Transcriptome Atlas of the Human Pancreas. *Cell Syst*. 2016;3(4):385-394.e3. doi:10.1016/j.cels.2016.09.002

2. Hashimshony T, Senderovich N, Avital G, et al. CEL-Seq2: Sensitive highly-multiplexed single-cell RNA-Seq. *Genome Biol*. 2016;17(1):1-7. doi:10.1186/s13059-016-0938-8

3. Schmalbrock LK, Dolnik A, Cocciardi S, et al. Clonal evolution of acute myeloid leukemia with FLT3-ITD mutation under treatment with midostaurin. *Blood*. 2021;137(22):3093-3104. doi:10.1182/blood.2020007626

4. Dobin A, Davis CA, Schlesinger F, et al. STAR: Ultrafast universal RNA-seq aligner. *Bioinformatics*. 2013;29(1):15-21. doi:10.1093/bioinformatics/bts635

5. Smith T, Heger A, Sudbery I. UMI-tools: modeling sequencing errors in Unique Molecular Identifiers to improve quantification accuracy. *Genome Res*. 2017;27(3):491-499. doi:10.1101/GR.209601.116

6. Stuart T, Butler A, Hoffman P, et al. Comprehensive Integration of Single-Cell Data. *Cell*. 2019;177(7):1888-1902. doi:10.1016/j.cell.2019.05.031

7. Genomics in the Cloud: Using Docker, GATK, and WDL in Terra - Geraldine A. Van der Auwera, Brian D. O’Connor - Google Boeken.

8. Md V, Misra S, Li H, Aluru S. Efficient architecture-aware acceleration of BWA-MEM for multicore systems. *Proc - 2019 IEEE 33rd Int Parallel Distrib Process Symp IPDPS 2019*. Published online May 2019:314-324. doi:10.1109/IPDPS.2019.00041

9. Tarasov A, Vilella AJ, Cuppen E, Nijman IJ, Prins P. Sambamba: fast processing of NGS alignment formats. *Bioinformatics*. 2015;31(12):2032-2034. doi:10.1093/BIOINFORMATICS/BTV098

10. McLaren W, Gil L, Hunt SE, et al. The Ensembl Variant Effect Predictor. *Genome Biol*. 2016;17(1). doi:10.1186/S13059-016-0974-4

11. Mayakonda A, Lin D-C, Assenov Y, Plass C, Koeffler HP. Maftools: efficient and comprehensive analysis of somatic variants in cancer. *Genome Res*. 2018;28(11):1747-1756. doi:10.1101/GR.239244.118

12. Gu Z, Eils R, Schlesner M. Complex heatmaps reveal patterns and correlations in multidimensional genomic data. *Bioinformatics*. 2016;32(18):2847-2849. doi:10.1093/bioinformatics/btw313

13. Serin Harmanci A, Harmanci AO, Zhou X. CaSpER identifies and visualizes CNV events by integrative analysis of single-cell or bulk RNA-sequencing data. *Nat Commun*. 2020;11(1). doi:10.1038/s41467-019-13779-x

14. Huang X, Huang Y. Cellsnp-lite: an efficient tool for genotyping single cells. *Bioinformatics*. 2021;37(23):4569-4571.
