# Supplementary material for: Longitudinal single-cell transcriptomics reveals distinct patterns of recurrence in acute myeloid leukemia
Source: Mol Cancer. 2022 Aug 19;21:166. doi: 10.1186/s12943-022-01635-4 (PMC9389773; doi:10.1186/s12943-022-01635-4)
Supplement: Supplementary file 2 — Additional file 2: Supplemental Table 1. Clinical information and sequencing details of the patients. [file 12943_2022_1635_MOESM2_ESM.pdf]

Patient ID	Gender	Age	Sample Source		Blast%		Enrichment		Time between Dx and Re
			Dx	Re	Dx	Re	Dx	Re	
**s220 *AET1-ETO***	Male	57	BM	BM	90%	NA	CD34	CD34	~ 10 months
**s914 *AML1-ETO***	Male	31	BM	BM	30%	NA	CD34	CD34	~ 10 months
**s232 *FLT3*-ITD***	Male	53	BM	BM	80%	90%	CD34	CD34	~ 8 months
**s292 *FLT3*-ITD**	Male	50	BM	BM	90%	60%	CD34	CD34	~ 14 months
**s2275 *FLT3*-ITD***	Male	33	BM	BM	95%	95%	CD34	CD34	~ 10 months
**s3432 *FLT3*-ITD***	Female	66	BM	BM	95%	91%	CD33	CD34	~ 12.5 months

* Treated with FLT3-ITD inhibitor: Midostaurin

Patient ID	Cell number after QC		Counts per cell (Mean)		Features per cell (Mean)	
	DX	Re	DX	Re	DX	Re
**s220 *AML1-ETO***	576	929	5 112	4 856	2 042	1 939
**s914 *AML1-ETO***	314	688	4 713	4 411	1 951	1 851
**s232 *FLT3*-ITD**	249	123	2 653	3 289	1 278	1 497
**s292 *FLT3*-ITD**	324	152	4 224	3 261	1 726	1 380
**s2275 *FLT3*-ITD**	309	281	3 530	3 681	1 328	1 457
**s3432 *FLT3*-ITD**	509	1 158	2 895	3 956	1 299	1 563

BM: Bone Marrow; NA: not available
