# Supplementary material for: Longitudinal single-cell transcriptomics reveals distinct patterns of recurrence in acute myeloid leukemia
Source: Mol Cancer. 2022 Aug 19;21:166. doi: 10.1186/s12943-022-01635-4 (PMC9389773; doi:10.1186/s12943-022-01635-4)
Supplement: Supplementary file 4 — Additional file 4: Supplemental Table 3. Characterization of FLT3-ITD at diagnosis and relapse. [file 12943_2022_1635_MOESM4_ESM.pdf]

| Patient ID | Time point | FLT3-ITD allelic ratio | ITD same Dx/Rel 1=yes, 0=no | ITD loss (=FLT3-ITD negative at rel) 1=yes, 0=no | ITD change at rel 1=yes, 0=no | switch (insertion site, length) 1=yes, 0=no | loss of min 1 clone at rel 1=yes, 0=no | gain of min 1 clone at rel 1=yes, 0=no |
|---|---|---|---|---|---|---|---|---|
| **s232** | Dx | 0,398 | | | | | | |
| | Re | 0,744 | 0 | 0 | 1 | 0 | 1 | 0 |
| **s292** | Dx | 0,659 | | | | | | |
| | Re | 0,71 | 0 | 0 | 1 | 1 | 0 | 0 |
| **s2275** | Dx | 0,988 | | | | | | |
| | Re | 26,312 | 0 | 0 | 1 | 1 | 0 | 0 |
| **s3432** | Dx | 0,617 | | | | | | |
| | Re | 0,325 | 1 | 0 | 0 | 0 | 0 | 0 |
